# Community-Engaged Air Monitoring to Build Resilience Near the US-Mexico Border

**DOI:** 10.3390/ijerph17031092

**Published:** 2020-02-09

**Authors:** Michelle Wong, Alexa Wilkie, Catalina Garzón-Galvis, Galatea King, Luis Olmedo, Esther Bejarano, Humberto Lugo, Dan Meltzer, Daniel Madrigal, Mariana Claustro, Paul English

**Affiliations:** 1 Tracking California, Public Health Institute, 555 12th St, Oakland, CA 94607, USA; alexa.wilkie@phi.org (A.W.); catalina.garzon@phi.org (C.G.-G.); galaking22@gmail.com (G.K.); DanMeltzer@gmail.com (D.M.); daniel.madrigal@phi.org (D.M.); 2 Comite Civico del Valle, 235 Main St, Brawley, CA 92227, USA; luis@ccvhealth.org (L.O.); esther@ccvhealth.org (E.B.); betomtz.lugo@gmail.com (H.L.); claustromariana@gmail.com (M.C.); 3 California Department of Public Health, 850 Marina Bay Pkwy P-3, Richmond, CA 94804, USA; paul.english@cdph.ca.gov

**Keywords:** air monitoring, air quality, citizen science, community air monitoring network, community engagement, community-based participatory research, community-engaged research, low cost, monitors, sensors, community resilience

## Abstract

Initiated in response to community concerns about high levels of air pollution and asthma, the Imperial County Community Air Monitoring Project was conducted as a collaboration between a community-based organization, a non-governmental environmental health program, and academic researchers. This community-engaged research project aimed to produce real-time, community-level air quality information through the establishment of a community air monitoring network (CAMN) of 40 low-cost particulate matter (PM) monitors in Imperial County, California. Methods used to involve the community partner organization and residents in the development, operation, and use of the CAMN included the following: (1) establishing equitable partnerships among the project collaborators; (2) forming a community steering committee to guide project activities; (3) engaging residents in data collection to determine monitor sites; (4) providing hands-on training to assemble and operate the air monitors; (5) conducting focus groups to guide display and dissemination of monitoring data; and (6) conducting trainings on community action planning. This robust community engagement in the project resulted in increased awareness, knowledge, capacity, infrastructure, and influence for the community partner organization and among community participants. Even after the conclusion of the original research grant funding for this project, the CAMN continues to be operated and sustained by the community partner, serving as a community resource used by residents, schools, researchers, and others to better understand and address air pollution and its impacts on community health, while strengthening the ability of the community to prepare for, respond to, and recover from harmful air pollution.

## 1. Introduction

Researchers and communities impacted by air pollution have demonstrated a growing interest in community-engaged research on air quality and related health outcomes in recent years [1]. Residents of communities near sources of air pollution, such as ports, freeways, and industrial facilities, are more likely to be exposed to high levels of air pollution and experience associated health outcomes [2,3,4]. The health effects of air pollution exposure include increased risk of respiratory illness, cardiovascular disease, preterm birth, and cancer [5,6,7,8,9]. Low-income people, children, the elderly, pregnant women, and those with pre-existing health conditions are especially vulnerable to air pollution exposures and these adverse effects. 

Environmental justice communities disproportionately impacted by air pollution have often been historically excluded from or marginalized in land use and environmental decision-making processes that determine where sources of air pollution are sited and how they are monitored and regulated [10,11]. Expanding community engagement in opportunities to collect and assess data to inform regulatory activities can help increase trust and effective collaboration between agencies and impacted communities. Meaningful engagement of community residents can also lead to enhanced environmental health literacy (defined as the understanding of the connection between environment and health and the ability to act on this based on that knowledge) [12,13]. The fields of community-based participatory research (CBPR) and citizen science (referred to in this article as community science) emphasize meaningful community participation in research and related decision-making [1,14,15]. These fields have provided an array of useful principles and tools to engage impacted residents in air quality research, monitoring, and regulatory efforts [16,17,18,19].

This article explores innovations in and outcomes of the application of CBPR and community science principles to the field of air monitoring, more specifically in the design, implementation, and use of a community air monitoring network (CAMN, a collection of low-cost air sensors distributed throughout a community) in Imperial County, California. This article will illustrate how the application of community-engaged research approaches, such as CBPR and community science (see Section 2), in academic and government air quality research efforts can enhance community awareness, knowledge, engagement, capacity, and resources (see Section 4). 

This article will also discuss how outcomes of this engagement can lead to increased community resilience for addressing health and environmental issues. For the purposes of this article, the term “community resilience” refers to the ability of a community to prepare for, respond to, withstand, and recover from adverse events such as natural disasters or changing environmental conditions [20]. For example, in California, worsening air quality has been identified as a localized impact of climate change that stands to disproportionately affect communities such as Imperial County that are already overburdened by air pollution and related health impacts [21]. This article explores how community engagement in air monitoring in Imperial County has contributed to strengthening core elements of resilience in this community for addressing air pollution, such as local knowledge, community networks and relationships, communication, governance and leadership, and health [20].

### Background

Imperial County is home to a primarily Latino population (84.6%) with some of the highest rates of unemployment and poverty in the nation [22]. The county is mainly desert and agricultural land, with a range of air pollution sources—such as field burning, the U.S.–Mexico border crossing, unpaved roads, and various industrial facilities—that contribute to exceedances of the California standard for particulate matter of 10μm or less (PM_10_) for time periods lasting over six months [23,24]. Historically, Imperial County far surpasses all other California counties as having the highest rate of both emergency visits and hospitalizations for asthma among school-age children [25], and a recent study suggests that respiratory impairment may be underdiagnosed in this population [26]. PM10 is related to increased respiratory disease, decreased lung function, and asthma attacks in susceptible individuals [27]. Awareness, understanding, and response to air pollution trends in the county have historically been hindered by the fact that there are only five regulatory PM monitors operating in a county that spans over 4000 square miles.

The Imperial County Community Air Monitoring Project (or “Imperial Project”) was a five-year project funded by the National Institute of Environmental Health Sciences (NIEHS) and conducted by Tracking California (formerly the California Environmental Health Tracking Program, a program of the non-profit Public Health Institute conducted in partnership with the California Department of Public Health) in collaboration with the local organization Comite Civico del Valle (CCV), the University of Washington, and other consultants [28]. Initiated in response to community concerns about air quality, the project brought together scientists, community advocates, and local residents to work toward a main goal: to better understand air pollution patterns in Imperial County in order to support actions to reduce air pollution exposures and improve health among residents. The Imperial Project used a community-engaged process to achieve its research and community priorities, which included the following: (1) assessing the feasibility of using low-cost, next-generation air sensors in a CAMN; (2) characterizing temporal and spatial patterns in air quality for the county; (3) using air monitoring results to inform individual and community exposure-reduction efforts; and (4) establishing the infrastructure and capacity for continued operation of the 40-monitor CAMN by community partners after the original research project ended. Previous articles on this project have described how the research process achieved its scientific aims [29,30,31,32]. The objective of this article is to share a conceptual model for understanding how methods used to engage the community in the Imperial Project were implemented to support different steps in the process used to establish a CAMN in Imperial County, California. We also discuss how the robust integration of community engagement in this research project has contributed to strengthening core elements of community resilience to air pollution in Imperial County, which indicates that the CAMN could contribute to longer-term reductions in exposures to air pollution and improvements in health outcomes.

## 2. Methods for Community Engagement

Effective ways of creating opportunities for meaningful community engagement in a research process are varied and context-specific [33]. Though there is no recipe for success, Wallerstein et al. (2008) delineate factors that can contribute to better understanding the outcomes of a community-based participatory research process, including mutual trust, group dynamics, shared governance and leadership, formal partnership structures, integration of local knowledge, and building community capacity. Many of these factors were incorporated into the selection, design, and implementation of methods for community engagement in the Imperial Project CAMN, as described here in Section 2. 

**Figure 1:**
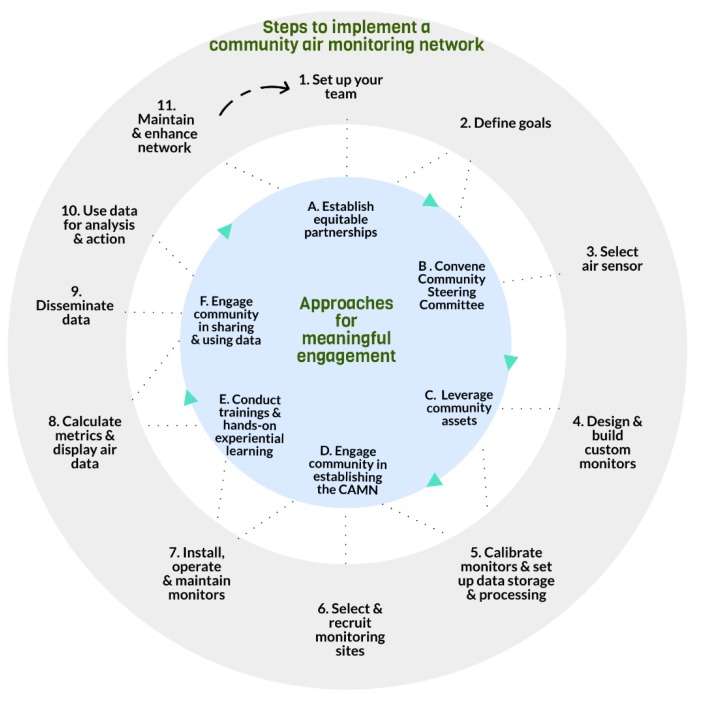
Model for meaningful community engagement in implementing the Imperial Project community air monitoring network (CAMN).

Figure 1 shows a conceptual model for community engagement in implementing a CAMN, from inception to maintenance, based on the Imperial Project. The outer ring of this diagram outlines key steps in the process of implementing a CAMN [34]. These steps include the following: (1) setting up your project team; (2) defining your goals; (3) selecting which sensor to use; (4) designing and building custom air monitors, if using them; (5) calibrating monitors and setting up data processing and storage; (6) selecting and recruiting monitoring sites; (7) installing, operating, and maintaining monitors; (8) calculating metrics and displaying air data; (9) disseminating data; (10) using the data for analysis and action; and (11) maintaining and enhancing the CAMN.

The inner circle of this diagram outlines key approaches or activities used to engage community partners or members in the process of developing, establishing, and using the Imperial Project CAMN. While the activities in the circle are placed most closely to the implementation steps to which they are most related (also denoted through the dotted lines), the approaches are sustained or repeated as needed to facilitate meaningful engagement as the project progresses through the steps in the outer ring. Thus, as indicated by its arrows, the inner circle is movable like a dial.

In the Imperial Project, an equitable partnership between researchers and the community-based organization was first established to meaningfully engage the directly impacted community in air quality research. This included sharing resources and decision-making power about the research process. Once mutual goals were set, the project team developed a structure for sustaining the engagement of a broader tier of community leaders to guide project design and implementation, known as a community steering committee (CSC). Regular CSC meetings convened by the project team were used to identify, leverage, and integrate local knowledge, social networks, and other community resources into the research and design process. In exchange, the project team contributed to building community capacity by providing technical training and expertise on air quality science, building and maintaining CAMNs, and other topics of interest for the CSC and other community members. The CSC and other community members contributed to developing community-relevant data displays to enable use of the data and engage in other communication strategies to share data with other target audiences. By the end of the project, the community partner played a lead role in maintaining the CAMN and ensured that any future enhancement or expansion activities were conducted using a community-engaged approach. 

The subsections below further describe how each of these community engagement approaches or activities were incorporated into and operationalized in the development, implementation, and use of the Imperial Project CAMN.

### 2.1. Establish Equitable Partnerships

The primary structure for equitably sharing decision-making, governance, and leadership in the Imperial Project was the formation and operation of a project team comprised of academic, community, and non-governmental partners. The partnership was facilitated by a decade-long relationship and prior collaborations between Tracking California and community partner CCV. Building upon this history, CCV was engaged in the project from its inception, identifying the need for a CAMN and participating in the development of the grant proposal to NIEHS. CCV’s director served as a formal co-investigator on the research grant. The project budget provided adequate and equitable funding for CCV to carry out project activities and participate in all activities related to project implementation and strategy. This approach aims to go beyond the more typical treatment of community partners as volunteers (with no or very limited compensation, such as with minor stipends) or as contractors/consultants (with more equitable financial compensation but little engagement in project decision-making).

Beyond shared funding, a key component of the partnership was ongoing communication to facilitate coordination, transparency, shared decision-making, relationship-building, and trust. Once the project was funded, the entire project team participated in a day-long strategy and team-building meeting to revisit project goals, objectives, and activities and to establish communication and decision-making processes. These in-person strategy meetings continued to be held about twice a year, along with monthly all-team phone meetings and bi-weekly phone meetings that focused specifically on community engagement activities.

### 2.2. Convene a Community Steering Committee

In addition to the formation of the project team, a community steering committee was established to provide guidance and decision-making for the project team and to serve as a formal structure for sustaining meaningful participation of community members distinct from CCV. The approximately 15 core members of the CSC included community advocates, teachers, high school students, representatives of local non-governmental organizations, and other concerned residents from throughout the county. Members were recruited to provide diverse knowledge, experiences, and perspectives to the project.

The CSC was formally convened at day-long, in-person meetings facilitated by the project team about twice a year, with additional engagement by CCV throughout the year. Members received modest stipends at the end of each meeting to facilitate their participation (e.g., to help offset any childcare or travel costs or time taken off from work). At the meetings, the CSC provided guidance and decision-making related to all aspects of the project, as shown in the conceptual model (Figure 1). This was facilitated by presentations and trainings, described below. The CSC also represented the project during community outreach and education activities, meetings with elected officials, and other activities involving interfacing with the broader community. The CSC was convened for the duration of the project, concluding in October 2018.

### 2.3. Leverage Community Assets

Leveraging community assets can build community capacity, increase the sustainability of the project, and strengthen relationships and build trust within the community. CCV and the CSC used their knowledge and networks to identify existing community resources, programs, and infrastructure that would align with, strengthen, and/or benefit from integration into the project. For example, we used CCV’s existing community-based web reporting system called IVAN (Identifying Violations Affecting Neighborhoods at www.ivan.org) as a primary way to store, process, display, and disseminate CAMN data. Through CCV’s existing relationships, we also partnered with schools to encourage the use and dissemination of CAMN data via their existing school air quality flag programs. Collaborating with local businesses, such as a local machine shop to create the enclosure for the monitor prototype, was another way in which community assets were integrated into the project. 

### 2.4. Engage Community Residents in Establishing the CAMN

In addition to convening CSC meetings, we designed specific activities to engage community members in the various steps of establishing the CAMN. One key activity was the selection of monitor sites, in which 45 community residents participated in a two-day process to learn about the project, identify potential monitor sites, collect data to assess the sites, and select final site locations [29]. CSC members also played a role in recruiting monitor sites, joining CCV in meetings with site representatives to help communicate about the importance of the study and convince them to host an air monitor. 

Another aspect of this approach was to be responsive to shifting community priorities and create opportunities for deeper community engagement. For example, CCV’s role in the original project plan did not include installing monitors. Due to personal interest, one of CCV’s outreach staff joined University of Washington in the initial monitor installations, participated in the calibration and validation of monitors [30], received training to assemble the monitors, and became the lead for all monitor installation and maintenance activities for the project. 

### 2.5. Conduct Trainings and Hands-on Experiential Learning

Establishing a common baseline understanding among community participants about the project background and activities can facilitate more meaningful, informed, and effective engagement and help ensure transparency and build trust with non-community partners. To achieve this, we conducted presentations and trainings on air quality and health, air monitoring technology and research, data collection and analysis processes, and research results. We also held trainings on conducting outreach about the project and developing community action plans. Finally, we established a youth environmental health internship program (YEHI) to engage local high school students to first learn about and then conduct outreach activities to peers, other community members, and government officials about air quality issues and the Imperial Project.

As noted above, we also developed hands-on experiential learning and training opportunities for community partners and participants to increase capacity to participate in this and other types of projects. This included participation and training in the research process (data collection during siting, monitor assembly, deployment, and maintenance). Project activities also included opportunities for CCV staff, CSC members, and youth interns to share their experiences and knowledge in new situations and with new audiences, such as meeting with elected officials and regulators; presenting at conferences and trainings; co-authoring manuscripts and textbook chapters; and sharing information with peers and other community members via presentations, tabling, and other community outreach methods. 

### 2.6. Engage Community in Communicating and Using CAMN Data

Data from a CAMN should be accessible, understandable, and useful for community residents. CCV and the CSC recommended that the main method of data dissemination should be a community website and notification system to provide alerts when air quality is considered harmful. To design this, we conducted focus groups with the CSC to determine how to calculate, display, and communicate the real-time Imperial Project CAMN data on the website. We assessed perceived data gaps and anticipated uses of monitoring data and priorities related to data quality, accessibility, and consistency with government data. We also got feedback on aspects of data communication, such as air quality metrics, categories and indices, terminology and labels, color schemes, units of time, and trends displays. Based on this input, we designed website mock-ups, used feedback to develop a beta website, and conducted user testing with the CSC prior to the public launch of the IVAN AIR website.

To increase awareness and use of the CAMN data, CSC members helped develop an outreach strategy, which included identifying target audiences, selecting dissemination methods, and crafting key messages. CCV led outreach activities, which included communication with federal, state, and local agencies and elected officials; schools; health clinics; and advocates and concerned residents. Some CSC members also volunteered to assist in conducting outreach about the CAMN at community meetings, churches, health fairs, media events, and more. Similarly, the YEHI interns also played a significant role in conducting outreach, particularly within their school communities. Finally, the CSC provided guidance on research activities, reviewing preliminary results of land use regression [31] and air pollution episodes analyses [32] and providing feedback on the interpretation, communication, and potential impacts of the results.

## 3. Methods for Evaluation

Evaluation priorities and activities for the community engagement in this project were developed with early CSC and project partner participation. We used a mixed methods approach, including minutes from project team and CSC meetings, participant evaluation forms collected at CSC meetings and other community events for the project, questionnaires administered to site selection participants, surveys conducted with schools engaged in air monitoring activities, evaluation forms distributed to participants following community meetings, trainings or data collection processes, and key informant interviews with project team members and CSC members at the conclusion of the project.

In 2018, Tracking California staff developed semi-structured interview questionnaires and contracted with an independent evaluator, the Survey Research Group (SRG), to conduct the key informant interviews, analyze interview responses, and develop a summary of results. SRG conducted individual interviews with nine CSC members still active at the end of the project (of the core 15 CSC members, five resigned after moving away from the area during the middle to end of the project timeframe). The remaining CSC member who did not participate was not available during SRG’s timeframe. CSC members were informed that their responses would remain anonymous, and they were not compensated to participate in the interviews. SRG also conducted three separate group interviews with staff from the key project partners (CCV, University of Washington, and Tracking California).

Interviews were conducted by phone with a notetaker present. SRG conducted qualitative data analysis of the interview notes and provided a final key informant interview report to Tracking California. Reporting of results from the interviews did not include identifying information such as names of interviewees, although quotations are attributed to the respondent type (CSC member or organization name). Tracking California then returned results of the interviews based on the SRG analysis to CSC members and project partners on separate calls held in September, November, and December 2018. The results of these interviews are described in Section 4.

A formal evaluation was conducted with YEHI interns, and the results will be described in another publication.

## 4. Results of Community Engagement on Resilience

The evaluation results indicate that the outcomes of robust community engagement in the Imperial Project described in Section 3 included strengthened relationships among impacted community stakeholders, increased knowledge about local air quality, increased capacity to engage in air quality science, and increased ability to take action to reduce exposure to air pollution. 

This section of the article discusses how the outcomes of this project contributed to strengthening collective resilience in this community to address air pollution in Imperial County, California. As noted in a review of 80 studies by Patel et al. [20], there is no single definition for “community resilience”. However, Patel et al. found nine core elements of community resilience to be common across the definitions: local knowledge, community networks and relationships, communication, health, governance and leadership, resources, economic investment, preparedness, and mental outlook. 

While much of the research that Patel et al. reviewed focused on community resilience in the face of disasters and other acute emergency situations, these nine elements can serve as a useful framework for examining community resilience to adverse environmental conditions, such as air pollution, more broadly. In this article, community resilience in the context of the Imperial Project refers to the community’s ability to act on, respond to, and recover from elevated ambient levels of air pollution as well as more acute episodes of poor air quality or air pollution events.

Key findings from the program evaluations to examine the impact of community engagement in the Imperial Project are described below for each of the nine elements of resilience as outlined by Patel et al. Some findings describe outcomes directly related to the engagement process, while others describe project outcomes that were formed, directed, or improved as a result of community engagement. The term “community participants” may refer to CCV staff, CSC members, YEHI interns, or any other community residents that participated in the project.

### 4.1. Local Knowledge

The element of local knowledge includes having a factual knowledge base and receiving training and education to increase knowledge, efficacy, and empowerment. The evaluation results indicate that participation in the Imperial Project increased knowledge for CCV staff and CSC members. Key factors that contributed to this knowledge included the CSC meetings, trainings, hands-on experiences, and CAMN data. 

CCV staff reported significant and diverse gains in knowledge, including increased understanding about the impacts of air pollution on health and increased ability to collect, access, interpret, and use air quality data via a CAMN. They felt that the opportunities for education, training, and hands-on experiences in operating low-cost air sensors, working with software and hardware, and conducting data management were critical to their ability to operate the CAMN at the end of the project. CCV staff also reported gaining skills and experience that increased their organizational efficacy. These included increased ability to (1) impact government decision-making through developing policy language, conducting advocacy, and communicating with legislators; (2) work with different types of institutions (e.g., government and academic) and require that any collaborations have effective partnerships, shared leadership and decision-making, and equitable funding; and (3) share their own knowledge outside of Imperial County by helping other communities establish CAMNs, trainings staff from regulatory agencies on community air monitoring, and presenting in scientific and environmental justice forums. 

Except for one CSC member who cited having previous scientific experience, the remaining eight CSC members reported gaining knowledge about the air monitoring process and how to interpret data. The formation of the CSC and the structure and educational content of the meetings were cited as key approaches in facilitating participation, engagement, and the exchange of information.

I was not just somebody who came to a meeting once in a while, randomly. I was a member of the steering committee and that was an important role not only for me but also my daughter. We had an opportunity to learn a lot in the process.

(CSC member, key informant interview report)

CSC members used their new knowledge to engage and educate other community members through actions such as establishing a high school environmental leadership club, writing news articles, speaking with coworkers, and advocating to decision-makers.

How can I talk to other people in a meaningful way about air quality if I don’t have that kind of education? It was first about acquiring that kind of knowledge for myself and my family.

(CSC member, key informant interview report)

I know more than I did before. I can bring valuable information to the newspaper [that I work for] and … it has been taken so seriously that…the newspaper donated more than 700 trees to improve our air quality all over the city and of course the border….

(CSC member, key informant interview report)

The real-time, community-level air quality CAMN data [35] were also cited as knowledge gained for the CSC and the broader community. The CAMN generated data during the last two years of the Imperial Project and continues to operate as of the writing of this article.

We have better information to show [decision-makers] and our community to advocate for them to take action at the local, state, and federal levels.

(CSC member, key informant interview report)

### 4.2. Community Networks and Relationships

Patel et al. found that well-connected community members and community cohesion were a key element in community resilience, citing trust and shared values as contributing factors. Evaluation results indicate that community networks and relationships were strengthened through participation in the Imperial Project, most notably through the convening of the CSC and through increased interactions with government and academic stakeholders. 

According to CCV staff, the CSC served as the nexus of the project, resulting in expanded networks that facilitated the project’s success (for example, CSC members led discussions with a school and a local utility company to install monitors at their locations). CCV staff felt that the existence of the CSC as a formal mechanism for community engagement increased community acceptance and trust in the project.

CSC members valued the new networks gained through their participation in the project, including relationships that developed between the CSC members and with non-CSC members (e.g., academic researchers, elected officials, and state and federal government representatives). Members appreciated the diverse expertise, experiences, and representation on the CSC, and CSC meetings were considered an effective way to engage in the project and network with others.

[We had] the opportunity to mingle. Having the freedom to have that voice was effective. None of the members remained silent. We all contributed in one way or another.

(CSC member, key informant interview report)

CCV’s participation also resulted in stronger professional networks. CCV staff reported increased visibility, credibility, and communications with government agency representatives, local and state elected officials, funders, academic researchers, and advocacy groups from other communities. They attributed this to CCV’s leadership role in the project, as well as the innovative nature of the project itself. In many cases, these entities reached out to CCV to learn more about the project and about local air quality issues. CCV staff noted an increase in the number of visits to Imperial County by government officials, raising the visibility of Imperial County’s environmental health concerns.

### 4.3. Preparedness

Preparedness involves having a plan in place from the individual to the governmental level to respond to a disaster event or, in the case of the Imperial Project, an air pollution event. Key factors for increasing community preparedness included leveraging community assets and community engagement in communicating and using CAMN data.

CCV and the CSC articulated the vision and helped execute a plan to (1) identify air pollution events via the CAMN; (2) communicate this information via the IVAN website and alert system; and (3) facilitate individual and community response by utilizing understandable, actionable, health-based messages for exposure reduction; conducting outreach and education activities; and collaborating with school flag programs. 

Preparedness among school communities was particularly strengthened. About one-third of the CAMN monitors were installed at schools, providing real-time data about school outdoor air quality. CCV conducted outreach and trainings with school staff to access and understand the CAMN data, establish a protocol for when to change colored flags (a visual marker corresponding to air quality), and adopt policies to respond to unhealthy levels of air pollution (such as keeping children indoors when air quality is poor). Of the 14 schools that had a CAMN monitor, 10 had operating school flag programs by the end of the project. YEHI interns also conducted outreach activities in schools to educate teachers and students about how to use the CAMN to take action during poor air quality events. By the end of their internship, most YEHI interns had increased their own use of air quality data to protect health. 

### 4.4. Communication

This element refers to the ability to communicate effectively during and after a crisis (such as natural or manmade disasters or other public health emergencies), which is facilitated by common meanings and understandings, opportunities to articulate needs, robust communication networks, diversity in communication mode and content, and accurate information. The Imperial Project resulted in increased communication about air quality and real-time air pollution events. A key approach for increasing communication was community engagement in communicating and using CAMN data.

CCV and the CSC played critical roles in shaping the communication and data display aspects of the CAMN. This included articulating community needs and barriers to accessing, understanding, and using air quality data; identifying priority methods for dissemination of CAMN data (website, alerts, school flag programs); assisting in the design and user testing of the IVAN website, air quality displays, and associated messaging; and designing and participating in community outreach activities to increase awareness of IVAN.

In the evaluation, CSC members and project partners specifically mentioned the CSC’s engagement in the website and messaging design process as an example of their unique contribution. However, respondents from both groups identified additional communication needs that were not met during the funded project period, including the development of additional audience-specific materials to engage decision-makers and the translation of more information into Spanish.

### 4.5. Governance and Leadership

This element includes the existence of infrastructure and services to respond to a situation, as well as the involvement and support of community and local government leaders in planning and response. Impacts on infrastructure and services are described above. Overall, community engagement in the Imperial Project has directly and indirectly increased community and government involvement and support in planning and response to air quality in the community, though CCV and CSC members had different perceptions of this.

CSC members emphasized the role of local government in addressing air pollution but cited challenges in engaging local leaders to take concrete action. In terms of their own involvement in planning and response to air pollution (beyond the Imperial Project activities), CSC members had mixed experiences. The project helped them to more effectively communicate with government leaders, but Imperial County’s geographic isolation from the state capitol was a barrier to their involvement in policymaking.

I spoke up at meetings on the record to elected officials and to directors of agencies…. I’m much [more] comfortable advocating for this topic now that I know a little more about it.

(CSC member, key informant interview report)

I would have been willing and able to advocate in Sacramento where a lot of decisions are made and policies are implemented, but we’re so far away that it’s difficult to sit at the table when these things are being discussed.

(CSC member, key informant interview report)

CCV staff reported greater involvement in government processes and felt they were more likely to have a “seat at the table” in government discussions. They also observed greater government engagement in community-led efforts. A major achievement cited by CCV was the passage of Assembly Bill 617 [36], jointly authored by California Assemblymember Eduardo Garcia, which mandated the development of a state Community Air Protection Program to reduce air pollution and improve health in impacted communities using several strategies, including community air monitoring [37]. CCV believed that the Imperial Project influenced AB617, because Assemblymember Garcia had engaged in the project by speaking at a CAMN launch event, sending staffers to attend CSC meetings and other project events, and working with CCV to author a separate bill on community science and air monitoring [38]. CCV also played a direct role in informing the implementation of AB617 by presenting at an informational legislative committee hearing on AB617 [39] and sharing lessons learned with California Air Resources Board (CARB) staff, who identified the Imperial Project as one of the successful models that would inform AB617 activities [40].

Government partners didn’t want to get involved before, but seeing the success of the work and the engagement of the community has increased their desire to be involved.

(CCV staff member, key informant interview report)

There is currently no formal mechanism for use of the CAMN data by government entities in planning and decision-making, although state and local engagement with community-generated air monitoring data continues to evolve as AB617 is implemented. CARB’s use of the CAMN data currently appears to be focused on assessing the data quality and performance of the CAMN sensors, although in one instance, the data were used by staff to examine spatial pollution gradients in the county. CARB also plans to launch a website that will display air quality data collected in AB617 communities, including by the Imperial Project CAMN [41]. To our knowledge, the local air district has not formally used the CAMN data and has only recently requested to access it. 

### 4.6. Health

This element refers broadly to a community’s understanding and ability to address health vulnerabilities in order to build resilience, including actions to maintain or improve health, deliver health services, or address mental health. Community participants’ understanding of air quality and health increased, but CSC members felt the project had not yet impacted health or local air quality on a community-wide scale.

I think we’re not at that step yet. That’s the next step- to improve the health by taking the identified problems to [politicians and health providers]. We have better information to show them and our community to advocate for them to take action at the local, state, and federal levels.

(CSC member, key informant interview report)

However, the development of the CAMN—and its integration with the IVAN alert system and school flag programs to reduce air pollution exposures—is an achievement that increases residents’ ability to protect their health.

### 4.7. Resources

This element refers to natural, physical, human, financial, and social resources distributed across a community. The Imperial Project increased physical, programmatic, human, and financial resources in the community. Key approaches included leveraging community assets, engaging the community in designing the CAMN, and conducting hands-on experiential learning.

The physical infrastructure of the CAMN, such as all monitors and equipment purchased via the research project, remains in the community. Community engagement in the site selection process helped to appropriately distribute monitors throughout the county, while training of CCV staff strengthened organizational capacity to ensure continued operation of the CAMN after the project’s completion.

The integration of community assets in the project presented opportunities to enhance existing infrastructure and skills. For example, in order to use CCV’s website to display the air monitoring data, their IT infrastructure was upgraded (e.g., new database and server). The website was also updated with a new design and additional features as part of this process. By coordinating with schools to integrate the CAMN data into existing or defunct school flag programs, CCV was able to (re)train school staff about the purpose and implementation of these programs and, for defunct programs, prompt their reinstatement. Increases in financial resources are described below.

### 4.8. Economic Investment

This element includes economic development, diverse economic resources, and proactive investments, all of which are vital for both response to and mitigation of future risks. Participation in the Imperial Project resulted in planned and unexpected economic investments in CCV and, by extension through CCV’s organizational activities, in the health of the broader community. Key approaches included establishing equitable partnerships, conducting trainings and experiential learning, and leveraging community assets. 

Appropriate sharing of funds is a critical component for establishing equitable partnerships. CCV reported that they received an equitable amount of the project funding. Through the project, two existing CCV staff were funded, and two new local positions were created for an air monitoring technician and the YEHI coordinator.

I think that’s where a lot of projects fail to be equitable where the community participation is undervalued and underappreciated (financially). This is not the case with this project.

(CCV staff member, key informant interview report)

CCV also secured new and diverse funding as a result of this project, citing their increased skills, co-investigator role, co-authorship in academic publications, and operation and stewardship of the CAMN as contributing factors. These funds included grants from state agencies and foundations, as well as subcontracts with academic researchers and other non-governmental organizations. This new funding supported the continued operation of the CAMN (to enable use of its data in research and other projects) and engagement in outreach, education, advocacy, and research projects aimed to reduce environmental exposures and improve health. The additional funding has enabled continuation of the two new CCV staff positions and allowed CCV to fund Tracking California and other consultants to provide technical support with ongoing CAMN operations and data analyses. 

### 4.9. Mental Outlook

The element of mental outlook includes hope, community pride, adaptability, and belief that one has the resources to deal with physically and emotionally distressing or challenging situations. Participation in the Imperial Project improved the community’s mental outlook. Instead of specific community engagement approaches, the overall achievements of the project and the community participants’ engagement and contributions to these successes were key factors in strengthened mental outlook.

CSC members and CCV staff reported feeling more empowered, hopeful, and prepared to make a difference. They expressed confidence in having the necessary tools to continue this work and meet their vision. Other community participants, such as YEHI interns and participants in the site selection activity, also reported feeling more prepared to plan and take action to protect their own health, advocate for clean air, and become more civically engaged.

CSC members were realistic about the extent of air pollution and the entrenched nature of environmental injustices in their community, but they were also positive in their assessment of the project’s impacts, expressing motivation and confidence about their ability to move forward.

It has empowered me by being able to express and defend our actions with good air quality information. For example, we were able to identify areas where air quality was worse and then able to identify why and where it was coming from. We were able to…bring those up to the proper agencies.

(CSC member, key informant interview report)

The biggest obstacle is still the ‘business as usual’ mentality of our largest economic drivers in our community- agribusiness and water and power companies and the county board of supervisors and their department.

(CSC member, key informant interview report)

The project has achieved its goals, but there are a lot more things that can be done…. We can’t stop right now. We cannot expect to solve this huge issue with this one project.

(CSC member, key informant interview report)

## 5. Discussion

Next-generation low-cost, portable air monitoring technology represents an opportunity for academic researchers, government agencies, communities, and concerned residents to innovate local air quality research [42,43]. The technology’s increased affordability and ease of use has enabled growing community engagement in air monitoring projects and garnered increased attention from academic and government researchers on the motivations, practices, and impacts of community air monitoring [44,45].

This case study describes approaches to and outcomes of community engagement in a community air monitoring research project in Imperial County, California. A central tenet of CBPR is that those most impacted by an issue being studied should be provided with substantive opportunities to be involved in and benefit from the research process. Similarly, community science seeks to leverage local knowledge by engaging lay people and community residents in collecting data and contributing to the scientific enterprise. Principles and tools from these fields can be applied in a project- and community-specific manner throughout the air monitoring research process, from the identification of research questions to the dissemination of findings. As described in this article, the concept of community resilience can also be useful in understanding how community engagement activities contribute to desired intermediate outcomes of a community air monitoring project, which may indicate that this can ultimately result in longer-term desired outcomes such as reductions in exposure and emissions or improved health outcomes.

### 5.1. Successes

This is the most community engagement and local interest in air quality I’ve seen… in the area.

(CCV staff member, key informant interview report)

Community engagement in the Imperial Project led to improved outcomes across the nine resilience elements. Some elements, such as local knowledge, community networks, and economic investment, had stronger gains while others, such as health, had more limited impacts. While we did not attempt to comprehensively assess the impact of specific community engagement approaches on each element, some relationships among the approaches and impacts emerged. Gains in some resilience elements were more directly related to approaches similar in theme, such as the element of community networks and relationships with the approach of convening a CSC, the element of communication with the approach of engaging the community in sharing and using data, and the element of local knowledge with the approach of training and hands-on experiential learning. In other cases, outcomes in resilience elements were not related to specific approaches but instead were related to the existence of the CAMN (such as the elements of preparedness and infrastructure) or the Imperial Project overall (such as the elements of mental outlook and governance and leadership), both of which were accomplished with significant community engagement. 

Several of the project’s community engagement approaches stood out as foundational to its success: establishing equitable partnerships, convening a CSC, and leveraging community assets. Several factors made the partnerships successful: (1) shared co-investigator duties formalized each organization’s commitment and accountability to the project, the other partners, and the funder; (2) equitable distribution of funds to support CCV’s participation underlined their importance as a lead organization; and (3) frequent project team meetings ensured transparency and engagement in project planning, coordination, and decision-making. The partnerships were also facilitated by a shared vision and commitment to the project, clear roles, feelings of mutual respect and trust, efforts to address issues of power and privilege, and awareness of one’s own biases and perspectives. Some challenges were also identified in the course of the partnership, including different work and communication styles, geographic distance between partners, cultural differences (including organizational and professional culture), and challenges in communicating about highly technical aspects of the project that can unintentionally limit transparency, causing confusion and mistrust that must be addressed.

Factors that contributed to the effectiveness of the CSC approach included (1) careful selection of dedicated community members that represented a diversity of locations, experiences, and ages; (2) communicating clear roles, expectations, and commitments for participation; (3) committing to day-long CSC meetings for more in-depth presentations and discussions; (4) establishing a group identity and camaraderie that allowed for open and safe communication, respectful disagreement, and mutual exchange of ideas; (5) holding trainings on core scientific principles related to air monitoring to establish a common knowledge base and facilitate informed engagement; and (6) offering meeting stipends to offset any financial impacts of participation and as a gesture of appreciation of the CSC’s time and effort. Challenges that were encountered in facilitating the CSC included maintaining engagement in between meetings and the loss of CSC members who moved out of the area to attend college or for new jobs.

I think that the communities were well represented [on the CSC], and we worked really well with [the CSC meeting] structure. It also has to do with the way the training was presented. The people leading us were very well-prepared in terms of managing our time and preparing agendas for those meetings. When the logistics are very well-planned there aren’t as many issues.

(CSC member, key informant interview report)

Finally, leveraging community assets was critical to the project’s success. The existing IVAN website and school flag programs were critical to the establishment and sustainability of the CAMN as a community resource. These assets were also strengthened through this project (e.g., new servers for IVAN, school staff re-trained on the flag program), increasing their sustainability as well. Because the website and flag program were already in place, these mechanisms for data dissemination did not rely on project funding and were more easily sustained after the project ended. However, leveraging community assets was not by itself sufficient for sustaining the costs of maintaining the CAMN. CCV’s ability to secure diversified funding has so far ensured the continued operation of the CAMN (and limited continuation of YEHI), covering costs related to equipment, staffing, and technical consultants including Tracking California. This has also resulted in an unexpected but appropriate and equalizing shift in CCV and Tracking California’s fiscal relationship. Whereas Tracking California was the recipient of the original NIEHS funding to establish the CAMN and YEHI, now CCV has the financial resources and power to choose if and how to engage others in the continuation of these efforts. At the time of writing, the CAMN continues to operate the 40 monitors and will be adding 15-20 more monitors in an adjacent county.

This is the most successful project that I’ve been involved with. This one has had a national and perhaps even an international impact.

(CCV staff member, key informant interview report)

### 5.2. Areas for Improvement

Early in the project, the CSC expressed the desire for the project to result in local policy changes to improve environmental and health conditions. Community participants took part in community action planning, but the larger goal of local policy change was not met within the project timeframe. This may be attributable to several factors, particularly the limited staff resources to devote to facilitating policy processes and the completion of the CAMN data analyses toward the end of the project, leaving limited time to plan a policy response. Additionally, a lack of engagement from local government may have resulted in lost opportunities for collaboration. For example, the local air district expressed little interest in the project beyond receiving updates as part of the project’s technical advisory group, and the local health department did not respond to early efforts by the project team to engage them. Furthermore, there continue to be deliberations at the local, state, and federal level about how community-generated air monitoring data should be used by regulatory agencies and what data quality standards should be met. The absence of guidelines may have also contributed to the lack of local government response to the CAMN data.

Despite these challenges, strong engagement and support by CARB, the demonstrated ability of the CAMN to produce scientifically rigorous community air quality data, and the implementation of AB617 has compelled the local air district to now pay closer attention to the CAMN and engage with CCV and the CSC. Most significantly, the implementation of AB617 was strongly informed by this project and has resulted in mandated activities and additional funding for the local air district to address poor air quality in parts of Imperial County, including the required development of local emissions reduction measures with community input.

### 5.3. Limitations

SRG did not interview all CSC members. The evaluations were not designed to measure resilience among the community participants or attribute this to specific engagement approaches, nor did they attempt to examine impacts of the project on the broader community. While outcomes of community engagement have been documented under the nine elements of resilience, a longer timeframe and a more rigorous and expanded evaluation would be needed to assess if and how community engagement in this project has contributed to increased community-wide resilience to air pollution.

### 5.4. Adaptability and Future Directions

This article described the successful application of community engagement practices in developing and sustaining a community-operated CAMN as part of an air monitoring research project. The project model and specific engagement methods may be applied in other air monitoring projects, though further study is needed to determine how these practices may fit and interact with a given community’s unique air monitoring needs, assets, funding resources, and timeline.

## 6. Conclusions

This article describes methods used to engage community members in an air monitoring research project and the impact of that engagement on community resilience. This case study demonstrates that communities have expertise and assets to contribute substantially to air monitoring research projects when they have equitable leadership roles and diverse mechanisms for meaningful engagement. 

Community engagement approaches can improve community resilience both directly and indirectly, such as when applied to establish community infrastructure, such as a CAMN. Although there is no “one-size fits all” set of approaches to community engagement, the fields of CBPR and community science offer many tools that can be appropriately and effectively used with the guidance and leadership of a community partner.

While community-engaged air monitoring projects may require substantial funding, time, and diverse team expertise, they can result in improved project outcomes and strengthened community capacity, sustainability, and resilience to address environmental and health conditions.
